# Health information-seeking behavior among Congolese refugees

**DOI:** 10.1371/journal.pone.0273650

**Published:** 2022-09-09

**Authors:** Elvis Longanga Diese, Eva Baker, Idara Akpan, Rushil Acharya, Amy Raines-Milenkov, Martha Felini, Arbaz Hussain

**Affiliations:** 1 Department of Pediatrics and Women’s Health, University of North Texas Health Science Center, Fort Worth, TX, United States of America; 2 Texas College of Osteopathic Medicine, University of North Texas Health Science Center, Fort Worth, TX, United States of America; PLOS ONE, UNITED KINGDOM

## Abstract

**Background:**

The purpose of this cross-sectional study was to determine the extent to which Congolese refugees seek health information, to identify and assess the resources used while exercising Health Information-Seeking Behavior (HISB), and to identify individual determinants that affect their HISB.

**Methodology:**

Building Bridges program participants who resided in Texas between 2017–2020, reported country of origin as Democratic Republic of Congo, and responded to HISB questions were included in this study. Four HISB questions asked about frequency seeking health information, preferred source and perceived trustworthiness of source, and frequency worrying about their health. Associations between HISB and sociodemographic factors (age, gender, education years, years in US, proficiency speaking English, marital status) were tested using Pearson chi-square or Fisher’s exact tests (α≤0.05).

**Results:**

Most participants (59%) reported seeking health information sometimes. Less than half (44%) of participants identified doctors as their preferred source of health information, Twenty-five percent relied on family, friends, and community leaders, and 23% used media sources. Doctors were identified as the most trustworthy source (71%), family and friends were the second highest trusted source (25%), whereas media sources were the least trusted (4%). Sociodemographic factors age (p = .02), gender (p < .01), and education years (p < .01) were the only significant predictors of preferred information sources. Conversely, those residing in US <5 years were more likely to seek health information more frequently (p = .01). The majority of participants did not worry about their health, and it was not significantly associated with source or frequency of seeking health information.

**Conclusions:**

The high trust in doctors represents an opportunity for healthcare professionals to educate and address individual barriers contributing to refugees’ underutilization of preventive care services such as routine immunizations and preventive health screenings.

## Introduction

Health information-seeking behavior (HISB) is generally defined as an active process of acquiring health-related information. This may include gathering knowledge about health status, illnesses, risks factors, and preventive measures or remedies [1, 2].

Several factors affect the need for individuals to seek health-related information. These include personal health concerns resulting from illnesses, thirst for knowledge, or media coverage of health-related information [3]. Rather than being a passive exposure to health information, HISB is an active and goal-oriented process during which individuals react and make an effort to acquire health-related knowledge [4].

Health information is important in decision-making and health promotion [5]. Although there is vast information on health, disparities persist among the populations based on an individual’s knowledge base and HISB. Low health literacy impacts how information is sought and understood and is a barrier to effective health care management [5, 6].

Engaging in HISB presents numerous benefits for individuals as they increase their health-related knowledge. Access and use of appropriate health information are associated with increased personal engagement to healthcare management, early diagnosis of diseases, better health-seeking behavior, disease control, medical treatment compliance, and satisfaction enhanced patient-provider shared decision-making, and overall better health outcomes [6, 7].

Every year, thousands of refugees from various countries around the world are resettled in the U.S. refugees themselves have little control over the resettlement process. They often find themselves in host countries that have few to no similarities with places of origin in terms of culture, language, or healthcare system. These differences contribute to low health literacy and have been found to interfere with healthcare delivery as refugees and immigrants are unable to navigate the complex healthcare systems of host countries [8, 9]. There is extensive literature on the strong relationship between low health literacy and poor health outcomes among individuals from culturally and linguistically diverse population groups [10–12]. Previous studies on refugee populations found that although refugees trust health professionals the most to give correct health information, they often turn to less reliable sources, such as friends and family, for their health information needs. Barriers such as language and limited accessibility to credible health information have been suggested as possible reasons for this behavior [13].

Congolese refugees are currently the largest refugee population arriving to the US since 2016 [14]. In the 2019 fiscal year alone, refugees from the Democratic Republic of Congo (DRC) comprised of about 43% of all refugee admissions in the United States, up from 35% in 2018 [14, 15]. Since 2016, more than 46,000 Congolese refugees have been resettled in the US [14], most of whom arrive to the states of Texas, Arizona, Kentucky, New York, and Colorado [16].

Congolese refugees mainly originate from the eastern part of the DRC where decades of armed conflicts have led to millions of deaths, vast displacements of populations, and unprecedented humanitarian crises [16–19]. Congolese refugees wait several years in refugee camps, mainly in Rwanda, Uganda, Tanzania, Burundi, and Kenya, before being resettled in resettlement countries [16, 20].

Although refugees from the DRC are multilingual and belong to various ethnic groups, most of them cannot understand, speak, or write English. The DRC has four national languages (Lingala, Kiswahili, Kikongo, and Tshiluba). According to the United States Refugee Admissions Program (USRAP) many speak Kinyarwanda because they are from the eastern DRC boarding Rwanda. Low literacy is another challenge among Congolese refugees. It is estimated that 70% of adults have never completed high school, and those entering the refugee program have not had access to secondary education [15, 16].

There is a significant disease burden on Congolese refugees as a result of a high prevalence of both communicable and non-communicable diseases [16]. In addition to extensive pre-departure and presumptive treatments, resettled Congolese refugees in the US have a need to be screened for a range of health conditions including chronic viral hepatitis, tuberculosis, HIV, diabetes, hypertension, and mental health [16, 21].

## Determinants of HISB

Accessibility and trust in a source influence the preference for where individuals seek their health information [1]. Traditionally, doctors and qualified health care professionals are the main reliable sources of health information. However, technological advancement has contributed to seeking other sources of health information, including the internet, to satisfy health needs [6].

Individual and contextual determinants influence how individuals seek health information. Demographic, psychosocial, and cultural factors are some of the individual determinants of HISB documented in the literature. One’s age, literacy level, ethnicity, gender, income, language, and length of residence are strong predictors of HISB [6, 11, 22, 23]. Other individual determinants of HISB include health anxiety, perceived self-efficacy, and perceived norms [6]. Contextual factors, such as community social capital and media advocacy, play an important role in the availability and accessibility of health information [6].

## Research purpose

The purpose of this cross-sectional study includes the following: (1) to determine the extent to which Congolese refugees seek health information; (2) to identify and assess the resources Congolese refugees use when exercising HISB; and (3) to identify individuals determinants that affect HISB among Congolese refugees. Identifying factors influencing HISB and the trusted sources of health information used by Congolese refugees may help clinicians and public health professionals to use appropriate channels and methods to affect health-seeking behavior change and improve the overall health of Congolese refugees. Understanding how refugees connect and engage with health information is important for designing targeted health interventions and reducing health care costs, particularly for local hospitals that carry the burden of non-indicated health-seeking behavior.

## Model

**Fig 1:**
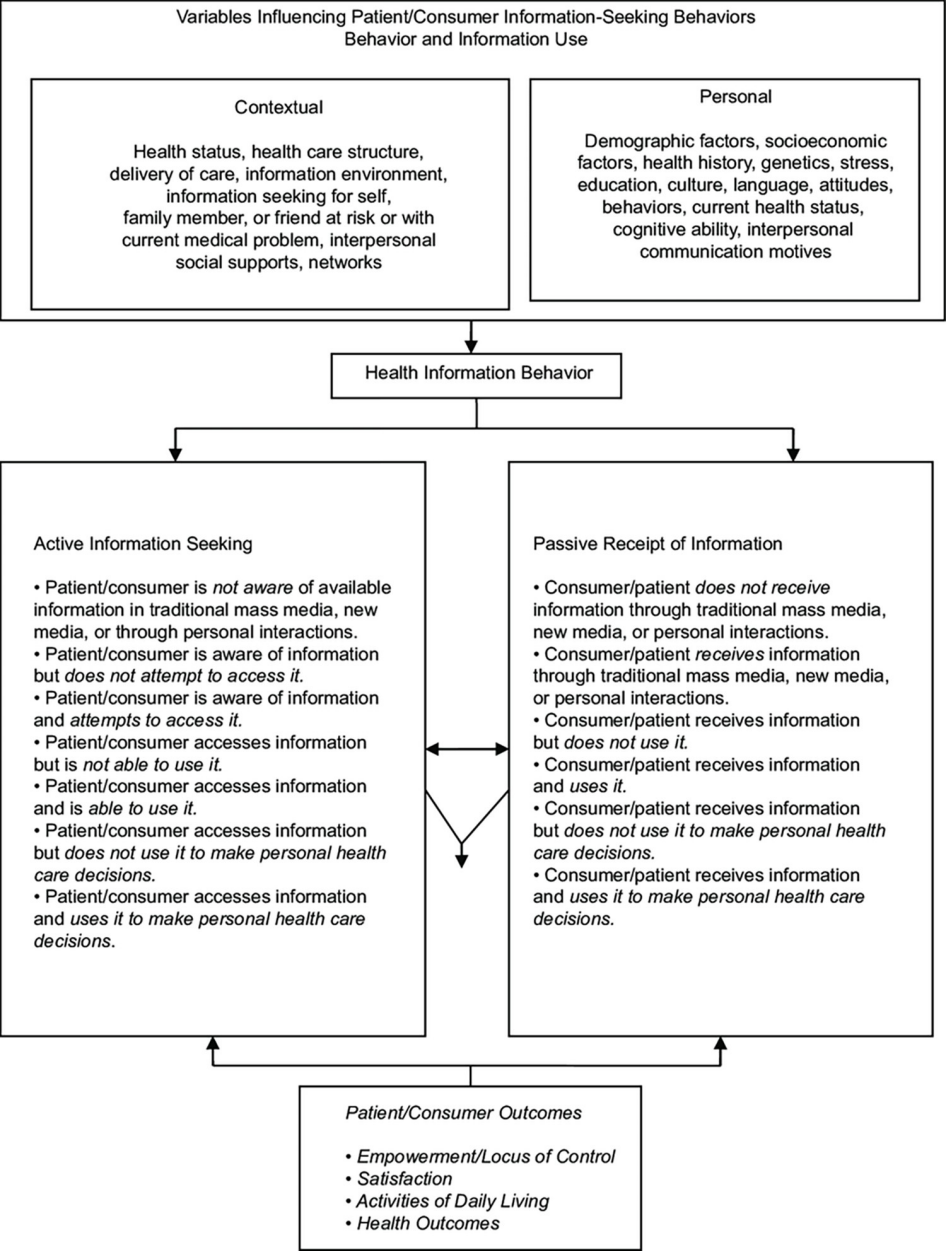
Longo’s expanded model of health information seeking behaviors [24].

This study examines the personal and contextual determinants of HISB following Longo’s model of health information-seeking behaviors [24]. Longo’s model (Fig 1) recognizes that individual (demographics, education, socioeconomic status, culture, language, stress, current health status, etc.) and contextual (information environment, accessibility to healthcare, and health information) factors can impact how information is sought and the preferences for health information sources. Individuals with higher literacy or education level are more likely to be active information seekers and have access to a variety of information sources including online sources [24]. Individuals with poor literacy are unable to adequately comprehend health information and are less confident in initiating conversations with their health care providers to discuss their health needs [25]. This model is appropriate for this study because it takes into account cross-cultural communication and health literacy concerns affecting HISB.

## Methods

Data from this study were collected as part of the Building Bridges Initiatives (BBI) funded by the Cancer Prevention Research Institute of Texas through the University of North Texas Health Science Center. The Building Bridges Program started in 2014 using a community health worker model to engage and provide male and female refugees 18 years and older and residing in Fort Worth, Texas, with culturally and linguistically appropriate cancer prevention education. Subsequent navigation to cancer prevention screenings for cervical cancer (including HPV vaccination), breast cancer, Hepatitis B and C was provided for eligible participants. CDC guidelines were used to determine eligibility for cancer screenings and vaccinations. The BBI employed a convenience sampling approach to recruit participants as most refugee groups were hard-to-reach for study participation. Participants were actively recruited into the Building Bridges Program by community health workers (CHWs) during outreach events at predominantly refugee churches and mosques, community education events, as well as in community locations where resettlement agencies initially resettle refugees. Each CHW used recruitment methods they felt most comfortable with and effective in their cultures. Additionally, they used their own social networks and encouraged “word-of-mouth” recruitment, whereby participants refer others to the program.

Participants served through the BBI represent adults who reported 24 different countries of origin. All participants completed a baseline assessment to identify needs and guide BBI cancer prevention service activities. Questions included in the BBI baseline survey were adapted from existing pre-validated questions. Due to the uniqueness of our study population, the final questionnaire was validated through a pilot study where it was administered to potential participants as well as to community leaders for consistency and appropriateness. Questions included sociodemographic information (17 questions), health insurance status (6 questions), health behaviors (smoking and alcohol—7 questions), trauma history (2 questions), and previous health diagnosis (1 question). In addition, questions were included on participant understanding, use, and experience using cancer prevention screenings and HPV vaccine (11 questions). In 2018, colon cancer screenings (3 questions) were similarly added as was HISB questions (4 questions). The complete assessment was 51 questions and could be completed in one or more home visits. Baseline assessments were administered in face-to-face encounters by trained community health educators from the refugee communities.

For purposes of this current study, we abstracted data pertaining to health information-seeking behavior and sociodemographic status (gender, age, income, level of education, English proficiency, marital status, and the number of years spent in the US) from the baseline assessment and restricted analysis to refugees from the Democratic Republic of the Congo only. Data abstracted from four questions about HISB included the following: “How often do you read or seek health information?” with the options *Not at all; Very little; Sometimes; A lot; Always*. A follow-up question “Where do you find most of your health information?” addressed the source of health information with the response options: *Doctors; Health educators; Family; Community leaders; and Others* with a write-in option to allow for a broad range of responses. The third question asked participants if they trusted their source of health information. Answer choices for this question included: *Yes; No; Sometimes*. The last question asked the following: “In the past month, how often do you worry about your health?” Answer options included the following: *Not at all; Rarely; Sometimes; A lot*.

Bivariate analysis was conducted between participant’s primary source of health information and selected covariates. Primary sources of health information were categorized at Doctor, Family and Community (inclusive of family, friends, and community leaders), and Media (inclusive of internet, google, facebook, YouTube, television, radio, and fliers). Responses with sources unspecified (n = 4) or with more than one information source identified (n = 4) were excluded from bivariate analysis. Selected covariates were categorized as binary due to small sample size. Pearson’s Chi-square test and Fisher’s Exact test were conducted between HISB and sociodemographic factors using a cutpoint of ɑ < 0.05 to determine statistical significance. Subsequent post-hoc analyses were performed for statistically significant associations between sociodemographic factors and the three major categories of health information using a Bonferroni correction (ɑ < 0.017). All descriptive and statistical data analysis were conducted using the Statistical Analysis Software (SAS 9.4, Cary, North Carolina).

Power calculations were performed using G*Power 3.1.9.7. With the 89 participants used in bivariate analysis between source of health information and sociodemographic factors, we achieved 70% power to determine an effect size of w = 0.3, but only 12% power to detect smaller effects size (w = 0.1), assuming an error probability of 0.05 and 2 degrees of freedom.

The North Texas Regional Institutional Review Board of the University of North Texas Health Science Center approved all study protocols. All study participants were provided with written consent forms in the language of their preference. Languages used by the study population included English, French, Kiswahili, Lingala, and Kinyarwanda. Key personnel read the consent form to participants unable to read by themselves. Written informed consent was completed by all study participants prior to enrollment into the BBI program.

## Results

A total of 101 Congolese refugees were selected for the analysis. As shown in Table 1, the majority of the participants (66%) were females and 51% were younger than 40 years of age. Overall, 79% of participants have spent less than 5 years in the US and 59% of participants have 10 years and more of formal education. However, 80% of the participants reported either *speaking English Not at all or Not well* (Table 1).

**Table 1:** Sociodemographic characteristics of Congolese building bridges participants, January 2018 –March 2020 (n = 101).

	Total
	n (%)
Gender	
Female	67 (66.3)
Male	34 (33.7)
Age (years)	
18–29	18 (17.8)
30–39	34 (33.7)
40–49	28 (27.7)
50–59	13 (12.9)
60 or above	8 (7.9)
Are you married?	
Yes	61 (64.2)
No	2 (2.1)
# of school years attended for formal education	
Less than 10 years	29 (30.2)
10 years or more	24 (25.0)
How well do you speak English?	
Not at all / Not well	81 (80.2)
Well / Very well	20 (19.8)
Years lived in US	
Less than 5 years	80 (79.2)
5–9 years	19 (18.8)
10 years or more	2 (2.0)

Table 2 shows that the most commonly cited source of health information was doctors (44%), a quarter (25%) sought health information from family, friends, and community leaders, and only 23% used online and other media sources. Four percent of participants reported using more than one primary source of health information. Health educators were not a key source of health information for this group (0%).

**Table 2:** Health seeking behavior of Congolese building bridges program participants, Fort Worth, Texas, January 2018 –March 2020 (n = 101).

	Total
	n (%)
Where Do You Find Most of Your Health Information?	97 (96.0)
Doctors	43 (44.3)
Family	19 (19.6)
Community leaders	1 (1.0)
Health Educators	0 (0.0)
Others	30 (28.9)
Internet = 15*	
Television = 5	
Friends = 4	
Radio = 1	
Fliers = 1	
Unspecified = 4	
>1 primary health information source	4 (4.1)
How Often Do You Seek Health Information	101 (100.0)
Always / a lot	3 (3.0)
Sometimes	60 (59.4)
Very little	28 (27.7)
Not at all	10 (9.9)
Do You Trust the Source?	95 (94.1)
Yes	61 (64.2)
Sometimes	32 (33.7)
No	2 (2.1)
Worry about your health?	96 (95.1)
A lot	29 (30.2)
Sometimes	24 (25.0)
Rarely	16 (16.7)
Not at all	27 (28.1)

On the frequency of seeking health information, the majority of participants (59%) responded to seeking health information *Sometimes*. *Fewer participants were not frequent seekers of health information (*28% Very little; 10% Not at all). Only 3 reported seeking health information Always (n = 1) or A lot (n = 2). Regarding the trust placed on the sources of health information sought, most participants (64%) reported *Yes* to trusting their sources of health information, 34% reported they trust their sources of health information *Sometimes*, and only 2% did not trust their sources (Table 2).

Across three of the primary sources of health information (Doctors, Family and Community, and Media), Participants aged less than 40 years were more likely to seek health information primarily from doctors (59%) and through media sources (27%), but less likely from family and community (14%). Those 40 years and older similarly sought health information from doctors (38%) and from family and community (40%) but were less likely to use media sources (22%) compared to their younger counterparts.

For gender, women were more likely to seek health information from doctors (53%) compared to men (39%). Also, women sought more information from family and community (33%) compared to men (16%). For media sources, men were more likely than women to seek health information (45% versus 14%). With regard to the number of school years attended, participants with less than 10 years of formal education were more likely than those with more years of education to seek health information from doctors (65% versus 37%). Among individuals with 10 or more years of formal education, 36% sought health information from media sources against only 8% among those with less than 10 years of formal education. There were no significant differences by marital status, English proficiency, and the number of years lived in the U.S. Regarding the trust, 40 out of 43 (93%) participants who sought health information from doctors trusted the source. Among those who relied on online sources, only 2 out of 20 (9%) trusted them.

We observed a statistically significant association for age (p = 0.018), gender (0.004), and the number of years of school attended for formal education (p = 0.005) across all major groups of health information sources, as shown in Table 3. Further post-hoc analysis on significant associations of sociodemographic factors using ɑ criteria < 0.017 revealed age remained an important determinant only in the comparison between Doctor versus Family and Community (p = 0.005), but not with gender (p = 0.523) nor years of education (p = 0.267). Conversely, age, gender, and years of education were statistically significant when comparing Doctor versus Media (age p value = 0.003; gender p value = 0.005; and years of education = p value 0.001). Gender remained an important determinant when comparing Family and Community versus Media (gender p value = 0.003), while age and years of education did not (age p value = 0.04; years of education p value = 0.05, data not shown).

**Table 3:** Source of health information, by sociodemographic characteristics of Congolese building bridges participants, January 2018 –March 2020 (n = 89).

		Where do you find most of your health information?			
Characteristics	Total (n = 89)	Doctors (n = 43)	Family & Community (n = 24)	Media (n = 22)	p value**
	n (%)	n (%)	n (%)	n (%)	
Age					
Less than 40	44 (49)	26 (59)	6 (14)	12 (27)	0.018*
40 and above	45 (51)	17 (38)	18 (40)	10 (22)	
Gender					
Female	58 (65)	31 (53)	19 (33)	8 (14)	0.004*
Male	31 (35)	12 (39)	5 (16)	14 (45)	
Marital Status					
Married	49 (55)	24 (49)	14 (29)	11 (22)	
Not married	40 (45)	19 (47)	10 (25)	11 (28)	0.843
Years formal education attended					
Less than 10 years	37 (42)	24 (65)	10 (27)	3 (8)	0.005*
10 years and above	52 (58)	19 (37)	14 (27)	19 (36)	
How well do you speak English?					
Not at all/Not well	72 (81)	34 (47)	21 (29)	17 (24)	0.620
Well/Very well	17 (19)	9 (53)	3 (18)	5 (29)	
Years lived in US					
Less than 5 years	71 (80)	35 (49)	17 (24)	19 (27)	0.396
5 years and above	18 (20)	8 (44)	7 (39)	3 (17)	
Do you Trust the Source?					
Yes	56 (64)	40 (71)	14 (25)	2 (4)	<0.001*
Sometimes/No	32 (36)	3 (9)	9 (28)	20 (63)	
Worry about your health?					
A lot/Sometimes	21 (36)	8 (38)	9 (43)	4 (19)	0.307
Rarely/Not at all	38 (64)	20 (52)	9 (24)	9 (24)	

*p<0.05.

**Calculated using Pearson’s chi-squared test, and Fisher’s exact test when more than 20% of cells have expected frequencies < 5. Note: Twelve participants were excluded due to missing source of health information (n = 4), unspecified source (n = 4), or >1 primary health information source (n = 4).

For the frequency of seeking health information (shown in Table 4), only the number of years lived in the U.S. was found to be a significant determinant. Sixty-eight percent of participants with less than 5 years in the U.S indicated they sought health information always, *a lot*, *or sometimes*, compared to only 38% among those who have lived in the U.S. five years or more. While age, gender, and education years were significantly associated with sources of HISB, they were not with the frequency of seeking health information (Table 4). Those not married, fluent in English, but trusting health sources and worried about their health proportionally sought health information more often than their counterparts, but these differences were also not statistically significant. Being worried or not about their health was not significantly associated with the types of sources or the frequency of seeking health information.

**Table 4:** Frequency of seeking health information, by sociodemographic characteristics of Congolese building bridges participants, January 2018 –March 2020 (n = 99).

		How Often do you Seek Health Information?		
Characteristics	Total (n = 99)	Always (n = 1)/A lot (n = 2)/ Sometimes (n = 60)	Very Little (n = 28) / Not at all (n = 10)	Chi-square p value
	n (%)	n (%)	n (%)	
Age				
Less than 40	51 (52)	33 (65)	18 (35)	0.515
40 and above	48 (48)	28 (58)	20 (42)	
Gender				
Female	65 (66)	41 (63)	24 (37)	0.679
Male	34 (34)	20 (59)	14 (41)	
Marital Status				
Married	52 (53)	28 (54)	24 (46)	
Not married	47 (47)	33(70)	14 (30)	0.094
Years formal education attended				
Less than 10 years	41 (41)	26 (63)	15 (37)	0.757
10 years and above	58 (59)	35 (60)	23 (40)	
How well do you speak English?				
Not at all/Not well	79 (80)	47 (59)	32 (41)	0.388
Well/Very well	20 (20)	14 (70)	6 (30)	
Years lived in US				
Less than 5 years	78 (79)	53 (68)	25 (32)	0.012*
5 years and above	21 (21)	8 (38)	13 (62)	
Do you Trust the Source?**				
Yes	60 (65)	41 (68)	19 (32)	0.299
Sometimes/No	33 (35)	19 (58)	14 (42)	
Worry about your health?**				
A lot/Sometimes	24 (36)	14 (58)	10 (42)	0.572
Rarely/Not at all	43 (64)	22 (51)	21 (49)	

*p<0.05.

**proportions calculated by excluding missing.

## Discussion

This study presents new information on health information-seeking behavior among Congolese refugees. Results show that most Congolese refugees frequently seek health information. This finding is consistent with the increasing rates of health information-seeking in US foreign-born populations observed in the Health Information National Trends Survey [25]. Findings observed in this study indicate the majority of Congolese refugees seek health information from medical doctors. Congolese refugees were less likely to seek information from media sources and their family community. Only a quarter of them reported seeking information from the internet or from other media sources such as television or radio. This finding is not consistent with most studies that reported the internet as the dominant source of health information among foreign-born US populations [19, 25, 26]. The observed limited use of the internet as a source of health in our study can be explained by the fact that 80% of study participants either do not speak well or do not speak English at all. In addition, most Congolese refugees in this study primarily speak dialects or languages, such as Kinyarwanda, Kibembe, Lingala, or Swahili. There are limited resources available online in these languages.

On the sources of health information, significant differences were observed by age, gender, and the number of years of formal education among study participants. Being younger than 40 years of age was associated with seeking health information primarily from doctors, and to a lesser extent from media sources. On the other hand, older Congolese refugees equally seek health information from doctors and their family community, but least likely from media sources. Similar findings were observed in other studies where it was found that older individuals, especially among immigrant populations were more likely to seek health information from other sources, such as doctors, family, and friends, than online [20, 27].

Gender differences observed in our study show that most women seek health information from their family community while the majority of men sought advice from doctors. While several reasons could potentially explain the gender difference observed, it is important to highlight that in the context of this study, often men are decision-makers in most African families, including for women’s health issues [28, 29]. However, women’s predisposition to seek health information from family and friends and community leaders is not unique to Congolese refugees or other immigrant populations. Similar findings were reported in a Finnish population-based survey where it was observed that women received far more health-related information from family and friends than men did [30].

Another significant determinant of sources of health information found in this study is the number of school years attended for formal education. Individuals with less than 10 years of formal education were the least likely to seek health information from media sources and primarily relied on doctors as their informants. This finding is consistent with results from other studies suggesting that less-educated individuals seek health information from non-online sources [27, 31]. The implication of this finding is important as most Congolese adult refugees resettled in the US never had access to secondary education [16]. Healthcare providers serving Congolese refugees should therefore be mindful about spending time to educate their patients since they have limited access to other reliable sources of health information.

Regarding the frequency of seeking health information, differences were only observed in number of years lived in the US. Refugees with less than five years in the US sought health information the most compared to those with five or more years in the US. The difference could be explained by the need for health information created by the numerous medical evaluations, screenings, and immunizations that newly resettled refugees receive after their arrival as well as while overseas before their departure to the US [16, 32]. For example, Congolese refugees are screened for parasitic infections and communicable diseases such as tuberculosis and chronic viral hepatitis [16]; and those who screen positive are referred for treatment.

This study further revealed that doctors are the most trusted sources of health information among Congolese refugees even more so than for those who seek health information from their family community. This trust represents an opportunity for health professionals to educate refugees and address some of the individual barriers contributing to refugee’s underutilization of preventive care services such as routine immunizations and screenings for cancer and chronic diseases [32–35]. Media sources of health information were the least trusted and among the least used among study participants. This lack of trust and limited use of media sources could be attributed to the limited English proficiency and the limited access to the internet in refugees’ countries of origin.

## Limitations of study

There are limitations to this study. It is important to ask for the reasons that participants selected their source of health information and the level of trust they put in that source. Adding “why” questions to the study may lead to further understanding and more detailed results. Further research should be conducted on finding out why these answers were selected by this group. Another limitation of the study was that it was notably underpowered to detect small effects. Thus, significant associations observed should be cautiously interpreted as a tendency rather than a reliable measure of association.

Our use of the cross-sectional design may limit the generalizability of the findings. There could be recall-bias where participants could not recall which places or frequencies they had sought health information in the past. This design also represents one moment in time, and the participants may shift their answers throughout the year or over time.

Never the less, this study presents important information for improved methods of outreach and education between healthcare workers, resettlement agencies and others who provide health care and health information to Congolese refugees in their community.
